# Natural killer cells are required for the recruitment of CD8+ T cells and the efficacy of immune checkpoint blockade in melanoma brain metastases

**DOI:** 10.1136/jitc-2024-009522

**Published:** 2024-11-17

**Authors:** Christopher Fife, Jennifer Williams, Fiona James, Scott Gregory, Tereza Andreou, Ashley Sunderland, Clive McKimmie, Rebecca J Brownlie, Robert J Salmond, Samuel Heaton, Fiona Errington-Mais, Zarnaz Hadi, David R Westhead, Marlous Hall, Alexander Davie, Amber Emmett, Mihaela Lorger

**Affiliations:** 1 Leeds Institute of Medical Research at St James's, University of Leeds, Leeds, UK; 2 Cancer Research UK National Biomarker Centre, The University of Manchester, Manchester, UK; 3 Skin Research Centre, University of York, York, UK; 4 Leeds Institute for Data Analytics, University of Leeds, Leeds, UK

**Keywords:** Melanoma, Central Nervous System Cancer, Immune Checkpoint Inhibitor, Immunotherapy, Natural killer - NK

## Abstract

**Background** Brain metastases (BrM) affect up to 60% of patients with metastatic melanoma and are associated with poor prognosis. While combined immune checkpoint blockade of programmed death-1 (PD-1) and cytotoxic T-lymphocyte-associated protein 4 (CTLA-4) demonstrates intracranial efficacy in a proportion of patients with melanoma, the responses are rarely durable, particularly in patients with symptomatic BrM. The brain is an immune-specialized organ and immune responses are regulated differently to the periphery.

**Methods** Using our previously established two-site model of melanoma BrM with concomitant intracranial and extracranial tumors, in which clinically observed efficacy of the combined PD-1/CTLA-4 (PC) blockade can be reproduced, we here explored the role of natural killer (NK) cells in BrM, using functional studies, immunophenotyping and molecular profiling.

**Results** We demonstrate that NK cells are required for the intracranial efficacy of PC blockade. While both perforin and interferon gamma were necessary for the PC blockade-dependent control of intracranial tumor growth, NK cells isolated from intracranial tumors demonstrated only a limited cancer cell killing ability, and PC blockade did not alter the abundance of NK cells within tumors. However, the depletion of NK cells in PC blockade-treated mice led to tumor molecular profiles reminiscent of those observed in intracranial tumors that failed to respond to therapy. Furthermore, the depletion of NK cells resulted in a strikingly reduced abundance of CD8+ T cells within intracranial tumors, while the abundance of other immune cell populations including CD4+ T cells, macrophages and microglia remained unaltered. Adoptive T cell transfer experiments demonstrated that PC blockade-induced trafficking of CD8+ T cells to intracranial tumors was chemokine-dependent. In line with this, PC blockade enhanced intratumoral expression of several T cell-attracting chemokines and we observed high expression levels of cognate chemokine receptors on BrM-infiltrating CD8+ T cells in mice, as well as in human BrM. Importantly, the depletion of NK cells strikingly reduced the intratumoral expression levels of T cell attracting chemokines and vascular T cell entry receptors that were upregulated following PC blockade.

**Conclusion** Our data demonstrate that NK cells underpin the efficacy of PC blockade in BrM by orchestrating the "responder" molecular profile in tumors, and by controlling the intratumoral abundance of CD8+ T cells through regulation of multiple key molecular mediators of T cell trafficking.

**WHAT IS ALREADY KNOWN ON THIS TOPIC**

- Combined programmed death-1 (PD-1)/cytotoxic T-lymphocyte-associated protein 4 (CTLA-4) (PC) blockade demonstrates intracranial efficacy in select patients with melanoma and brain metastases (BrM) as well as in preclinical models, and this requires CD8+T cells. In contrast, the role of natural killer (NK) cells in antitumor immune responses as well as in the efficacy of immune checkpoint blockade (ICB) in BrM remain unexplored, and only a limited number of studies investigated the involvement of NK cells in ICB at extracranial sites.

**WHAT THIS STUDY ADDS**

- This study demonstrates that NK cells are required for the efficacy of combined PC blockade in BrM. The role of NK cells in intracranial ICB efficacy goes beyond direct cancer cell killing and involves reshaping of tumors into “responder” molecular profiles and recruitment of CD8+ T cells via intratumoral chemokine upregulation.

**HOW THIS STUDY MAY AFFECT RESEARCH, PRACTICE OR POLICY**

- By revealing NK cells as major players in the intracranial ICB efficacy in melanoma, this study is expected to stimulate future investigations into NK cell-focused therapies for the enhancement of ICB efficacy in BrM.

## Introduction

 Metastatic melanoma has a high risk of spreading to the brain, with up to 60% of patients developing brain metastases (BrM), which are associated with poor prognosis.^1^ Treatment options for melanoma BrM have been previously restricted to radiotherapy, surgery, and select targeted therapies, with immunotherapies targeting immune checkpoints being recent additions.^2^ Programmed death-1 (PD-1) and cytotoxic T-lymphocyte-associated protein 4 (CTLA-4) are immune checkpoints expressed predominantly on T cells and their inhibition with function-blocking antibodies can enhance antitumor T cell responses.^3 4^ Antibodies targeting CTLA-4 (ipilimumab) and PD-1 (nivolumab, pembrolizumab) have shown great promise for the treatment of different cancers, including melanoma.^5^ A combined targeting of CTLA-4 and PD-1 in treatment-naïve patients with melanoma with BrM demonstrated a 45–55% intracranial response rate, which was significantly higher as compared with the anti-PD-1 monotherapy (∼25% intracranial response rate).^6 7^ However, the response rate is significantly lower in patients with symptomatic BrM and the responses are rarely durable.^8 9^ It is therefore important to understand what drives the intracranial efficacy of immune checkpoint blockade (ICB), to enable the development of strategies for enhanced ICB activity in BrM.

The brain has an immune-specialized status with limited immune responses.^10^ In line with this, we have previously demonstrated a lack of intracranial ICB efficacy in mice bearing intracranial tumors only, while mice bearing concomitant intracranial and extracranial tumors demonstrated similar intracranial response rates to ICB as observed in patients with melanoma.^11^ Thus, mimicking the clinically observed intracranial ICB efficacy in preclinical models requires the concomitant presence of an extracranial tumor,^11^,^13^ which is needed to establish systemic antitumor immune responses following ICB.^11^ Of note, extracranial lesions, most commonly skin metastases, are also commonly observed in patients with melanoma BrM, and therefore this “two-site” model of BrM mimics the clinical situation.

Most studies to date have focused on T cells as mediators of antitumor response unleashed by ICB. While PD-1 and CTLA-4 on T cells are well explored, their role in other immune cell types is less well understood. In addition to T cells, PD-1 is also expressed in a subpopulation of tumor-infiltrating natural killer (NK) cells in mouse models and in human cancers. Its interaction with programmed death-ligand 1 (PD-L1) has been shown to inhibit NK cell antitumor function, including direct cytotoxicity and cytokine production, and this can be rescued through PD-1/PD-L1 blockade in preclinical models and patient-derived tissue.^14^,^17^ This implies PD-1 as an important immune checkpoint on NK cells. In comparison, the role of CTLA-4 in NK cells is less explored, with only a few studies reporting CTLA-4 surface expression on cancer-associated NK cells in mouse models^18^ and in patients with cancer.^19 20^ CTLA-4 expression on NK cells can be induced through NK cell activation with cytokines or target cells, and it inhibits the antitumor activity of NK cells.^18^,^20^ Recent studies have shown that NK cells are required for the efficacy of anti-PD-L1 monotherapy and combined PD-1/CTLA-4 (PC) blockade in subcutaneous tumor models,^14 21^ while the role of NK cells in ICB in the context of the immune-specialized microenvironment of BrM remains unknown.

We have previously demonstrated that intracranial efficacy of combined PC blockade requires systemic expansion of effector CD8+ T cells followed by their homing to BrM.^11^ In addition, we observed that NK cells were also required to extend the BrM-dependent survival following combined PC blockade in a two-site melanoma BrM model.^11^ In the present study we investigated how NK cells contribute to the intracranial efficacy of ICB in the brain. We focused on combined PC blockade because it shows superior intracranial efficacy in patients with melanoma as compared with monotherapies.^7^ We demonstrate a key role for NK cells that goes beyond cytotoxicity and direct cancer cell killing.

## Methods

### Animals and two-site BrM model

Six to 10-week-old male and female C57BL/6J, C57BL/6-*Prf1^tm1Sdz^*/J (Jax stock 002407) and B6.129S7-*Ifng^tm1Ts^*/J (Jax stock 002287) mice were purchased from Charles River Laboratories, UK, and maintained in a special pathogen-free facility in individually ventilated cages. Mice were acclimatized for 7 days prior to procedures. Sizzle nest and plastic domes were used as environmental enrichment.

B16 F1 or B16-OVA melanoma cells (2×10^5^) were subcutaneously injected on the flank to generate extracranial tumors. B16 F1 or B16-OVA cells (1×10^5^ or 1×10^4^) were intracranially injected into the striatum (2 mm right of the midline, 2 mm anterior of the bregma, 2.5 mm deep) to generate intracranial tumors. To exclude the time of day of implantation as a potential confounder, the order of implantation was alternated between the experimental groups.

Analgesics were administered prior to surgery and at 24 hours postsurgery. Mice were monitored daily (and up to three times per day in the last 2 days of experiments) for neurological symptoms, as well as undergrooming and reduced activity as general signs of compromised health. Throughout the experiment, extracranial tumor growth was quantified by caliper measurement by a blinded researcher. Intracranial tumor growth was quantified by non-invasive bioluminescence imaging using IVIS Spectrum and Living Image software (PerkinElmer). D-Luciferin (Tocris) was injected intraperitoneally and mice were imaged 12 min later. Mice were initially randomized into groups based on the litter identity and sex. Prior to the commencement of ICB treatment, and to ensure equal distribution of tumor burden across groups, mice were randomized into groups based on their intracranial bioluminescent signals to ensure comparable distribution of tumor burden across groups prior to therapy. Thus, the experimental unit was a single animal.

In some experiments, NK cell depletion was achieved through intraperitoneal administration of anti-Asialo-GM1 (Cedarlane) or anti-NK1.1 (Bio X Cell) at 100 µg/mouse every 4 days, as indicated. Anti-PD-1 (RMP1-14), anti-CTLA-4 (9D9), and immunoglobulin G (IgG) control (MPC11) were purchased from Bio X Cell and administered intraperitoneally at 200 µg per mouse, as indicated.

We compared the control group (IgG treated) to the therapy group (treated with a combined anti-PD-1 plus anti-CTLA-4) and a group of treated mice in which NK cells were depleted, as specified in the results section.

### Cell lines and in vitro culture

B16 F1 (B16) melanoma cells were obtained from the American Type Culture Collection. B16 OVA cells^22^ were generously donated by R Vile, Mayo Clinic, Rochester, Minnesota USA. Mycoplasma testing was routinely performed every 6 months. B16 and B16-OVA cells were cultured in Dulbecco’s Modified Eagle’s Medium (Sigma-Aldrich) supplemented with 10% fetal bovine serum (FBS) (Gibco), 1 × L-glutamine (Gibco), and 1 × penicillin-streptomycin (Gibco). Cancer cells were stably transduced with a Firefly luciferase (Fluc)-expressing lentiviral vector^23^ to generate B16/Fluc and B16-OVA/Fluc cell lines. YAC-1 cells were cultured in RPMI-1640 medium (Sigma) supplemented with 10% FBS (Gibco), 1 × L-glutamine (Gibco), and 1 × penicillin-streptomycin (Gibco).

### NK cell isolation and ^51^Chromium release assays to quantify NK cell cytotoxicity

Mice were treated with PC on days 4 and 6 post-intracranial implantation of B16-OVA/Fluc melanoma cells. NK cells were isolated from intracranial tumors 2 days after the last therapeutic dose. CD3+ T cells were depleted from cell suspension obtained following tumor dissociation via magnetic cell sorting using CD3e depletion kit (Miltenyi 130-094-973), followed by isolation of NK cells using CD49b (DX5) positive selection kit (Miltenyi 130-052-501).

B16-OVA or YAC-1 target cells were harvested and labeled with 100 µCi ^51^Chromium (^51^Cr)/10^6^ cells (PerkinElmer) for 1 hour at 37°C. Following incubation with ^51^Cr, target cells were washed three times in PBS by centrifugation and resuspended in RPMI-1640 containing 5% FBS and L-glutamine (complete RPMI-1640).

2,000 ^51^Cr-labeled B16-OVA or YAC-1 cells, respectively, were mixed with 40,000 isolated NK cells (1:20 ratio) in RPMI-1640 complete medium in round-bottom 96-well plates in a final volume of 150 µL and incubated for 4 hours at 37°C in a tissue culture incubator. Spontaneous release controls were established using target cells alone in a complete RPMI-1640 medium. Maximum ^51^Cr release was measured by culturing target cells alone in 1% Triton-X-100 (Sigma-Aldrich) in a complete RPMI-1640 medium. Following incubation, cells were pelleted and 50 µL of each supernatant was transferred to a LumaPlate (PerkinElmer) and left to dry overnight. The level of ^51^Cr in the supernatant was then measured using a MicroBeta scintillation counter (PerkinElmer) and the percentage of target cell lysis was calculated using the following formula (cpm: counts per minute): % lysis = 100 × (sample cpm—spontaneous cpm)/(maximum cpm—spontaneous cpm).

### RNA isolation, generation of sequencing libraries and mRNA-seq

RNA was isolated from dissociated tumor tissue using the RNAqueous Total RNA Isolation Kit (Ambion) as per the manufacturer’s instructions.

Sequencing library generation, messenger RNA sequencing (mRNA-seq) and data analysis were performed by Novogene. A total amount of 2 µg RNA/sample was used as the input material. mRNA was purified from total RNA using poly-A oligo-attached magnetic beads. After fragmentation, the first strand complementary DNA (cDNA) was synthesized using random hexamer primers, followed by the second strand cDNA synthesis. End repair, A-tailing, adaptor ligation, size selection, amplification and purification were performed.

Libraries were quantified with qubit and the size distribution was detected by Bioanalyzer. Quantified libraries were pooled. The clustering of the index-coded samples was performed according to the manufacturer’s instructions. Following cluster generation, the library preparations were sequenced on Illumina NovaSeq 6000 and paired-end reads were generated.

### mRNA-seq data analysis

Raw mRNA-seq FASTQ reads were quality checked and filtered using an in-house perl script, discarding reads with adaptor contamination, low quality (Q<5 constituting more than 50% of the read), and over 10% uncertain nucleotides (n>10%). Filtered paired-end reads were aligned to the mouse reference genome (Ensembl GRCm38.p6) using STAR (V.2.6.1d).^24^ Alignments were assembled to transcripts using Stringtie V.1.3.3b,^25^ and gene expression was quantified using featureCounts V.1.5.0-p3.^26^ Differential gene expression analysis was performed on raw gene expression counts using the DESeq2 R package (V.1.26.0).^27^ Genes were tested for differential expression by pairwise comparisons between the four experimental groups, each with three biological replicates. Differentially expressed genes were also identified between the CD8H group versus the three other groups. P values were adjusted by Benjamini-Hochberg correction, and genes with p value≤0.05 and false discovery rate (FDR)≤0.05 were identified as differentially expressed. Differentially expressed genes were hierarchically clustered by z-score log2 (FPKM+1) values and visualized as a heatmap using the pheatmap R package.

Differentially expressed genes were ranked by their log2 fold change and subjected to Gene Set Enrichment Analysis (GSEA) with the Kyoto Encyclopedia of Genes and Genomes (KEGG) reference database, using an online tool WEB-based GEne SeT AnaLysis Toolkit,^28^ with a FDR cut-off value of≤0.05 to identify significantly enriched pathways.

Genes enriched in the KEGG pathway “natural killer cell mediated cytotoxicity” (n=46) were selected for functional protein association analysis using the STRING database.^29^ The confidence threshold for edges between two nodes, representing shared protein–protein associations was maintained at the default level of 0.4 (medium confidence).

### Human BrM tissue

Clinical samples and associated clinical data were collected at the Leeds General Infirmary, The Leeds Teaching Hospitals Trust, and sourced from the Leeds Multidisciplinary Research Tissue Bank (REC reference: 20/YH/0103) and Leeds Neuropathology Research Tissue Bank (REC reference: 20/YH/0109).

### Statistical analysis

We used power calculations to determine group sizes, using bioluminescence signal intensity or immune cell percentages as a primary outcome measure.

Statistical analyses were carried out using GraphPad Prism V.10 (GraphPad Software). Outliers were identified using the ROUT method and removed. Shapiro-Wilk tests were used to assess the normality of data for each experiment. Differences in samples were determined using unpaired two-tailed t-tests (pairwise comparisons) and one-way analysis of variance with a pairwise comparison of median values (multiple comparisons) for normally distributed data and unpaired Mann-Whitney tests (pairwise comparisons) and Kruskal-Wallis tests with a pairwise comparison of median values (multiple comparisons) for non-normally distributed data. Data were plotted with error bars based on the standard deviation (SD). The number of biological replicates for each experiment is stated in figure legends alongside the type of analyses conducted.

Further methods can be found under online supplemental materials.

## Results

### NK cells are critical for the intracranial efficacy of ICB

**Figure 1:**
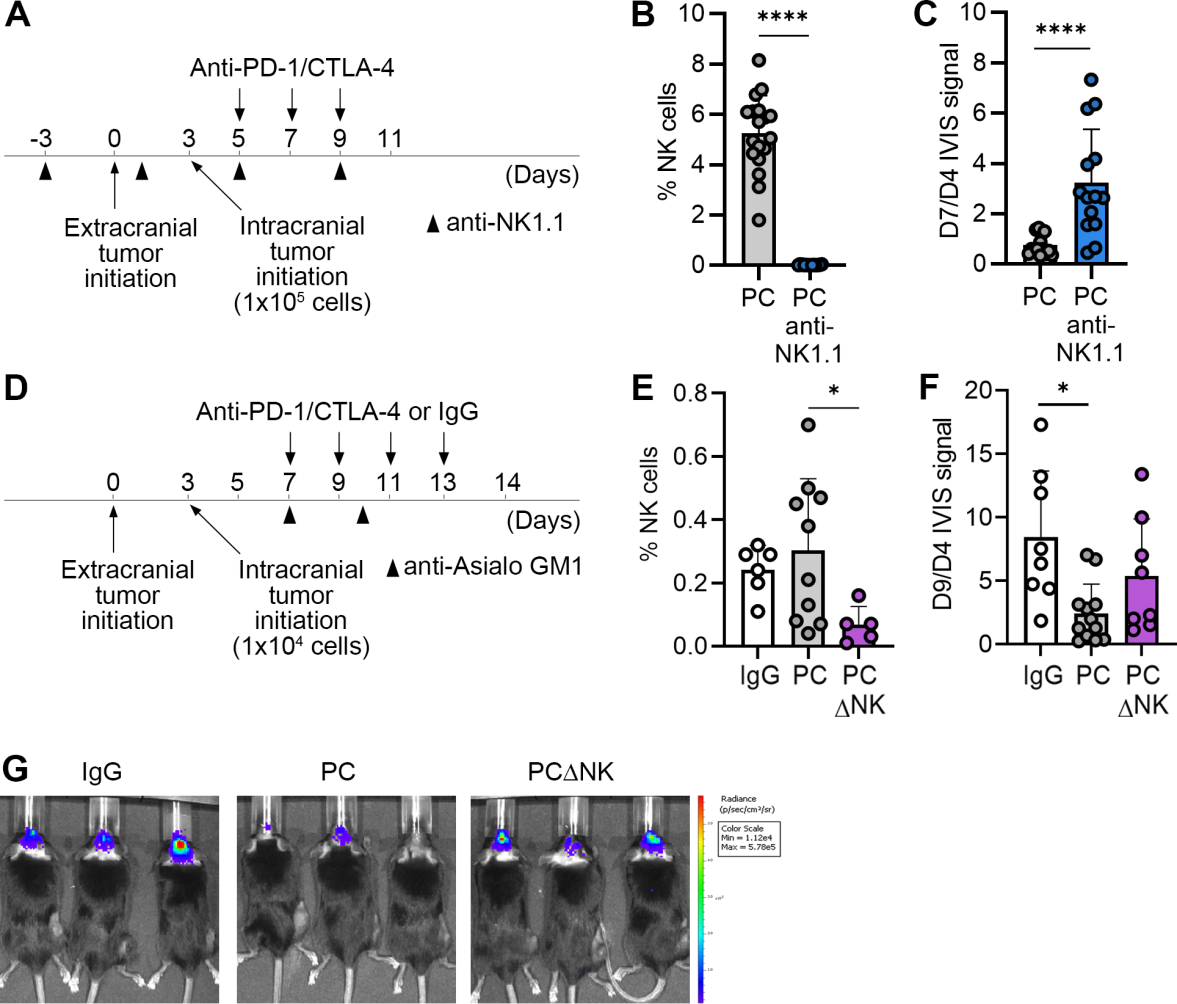
Depletion of NK cells results in a loss of intracranial ICB efficacy. (**A**) Experimental scheme for experiments shown in **B** and **C**. The timeline for implantation of B16-OVA (s.c.) and B16-OVA/Fluc (intracranial) cancer cells in the two-site BrM model, therapeutic schedule, and schedule for administration of anti-NK1.1 antibodies for depletion of NK cells are indicated. (**B**) Quantification of NK cells within intracranial tumors by flow cytometry (n=16/18 for PC and PC anti-NK1.1, respectively). Combined data from two independent experiments are shown. NK depletion in the PC anti-NK1.1 group (blue) was performed throughout the experiment, as indicated in **A**. (**C**) Quantification of intracranial B16-OVA/Fluc tumor burden via bioluminescence imaging (n=14/14 for PC and PC anti-NK1.1, respectively). Data from two independent experiments were combined. Fold change in bioluminescence signal intensity between days 7 and 4 post-intracranial cancer cell implantation, corresponding to days 10 and 7 of the experimental timeline (eg, pretreatment/post-treatment with PC) is shown. NK depletion in the PC anti-NK1.1 group (blue) was performed throughout the experiment, as indicated in **A**. (**D**) Experimental timeline for experiments shown in **E–G**, including schedule for administration of anti-Asialo-GM-1 antibodies for depletion of NK cells post-first therapeutic dose. (**E**) Quantification of NK cells within intracranial tumors by flow cytometry (n=6/10/5 for IgG, PC and PCΔNK, respectively). NK depletion in the PCΔNK group (purple) was performed after the first treatment dose, as indicated in **D**. (**F**) Quantification of intracranial B16-OVA/Fluc tumor burden via bioluminescence imaging (n=8/12/8 for IgG, PC and PCΔNK, respectively). Fold change in bioluminescence signal intensity between days 9 and 4, corresponding to days 12 and 7 of the experimental timeline (eg, pretreatment/post-treatment with PC) is shown. NK depletion in the PCΔNK group (purple) was performed after the first treatment dose, as indicated in **D**. (**G**) Representative bioluminescence images of mice from experiment following the timeline shown in **D**. Statistical differences were determined by Mann-Whitney test (**B and C**), one-way ANOVA (**E**) and unpaired Kruskal-Wallis test with pairwise comparison of mean values (**F**); *p≤0.05, **p≤0.01, ***p≤0.001, ****p≤0.0001. ANOVA, analysis of variance; BrM, brain metastases; CTLA-4, cytotoxic T-lymphocyte-associated protein 4; Fluc, Firefly luciferase; ICB, immune checkpoint blockade; IgG, immunoglobulin G; NK, natural killer; PC, PD-1/CTLA-4; PD-1, programmed death-1; s.c., subcutaneous.

We first confirmed by flow cytometry that NK cells can be detected in patient melanoma BrM obtained from two different patients (1.18% and 1.45% of intratumoral CD45+ cells, respectively), with majority of NK cells expressing the activation markers NKp46 (76.4% and 88.1% of NK cells, respectively) and CD69 (93.2% and 98.2% of NK cells, respectively) (online supplemental file 1A), as well as in intracranial tumors in the two-site BrM model established with B16 melanoma cells (online supplemental figure 1B). Using the same two-site BrM model, we previously demonstrated that depletion of NK cells with anti-Asialo-GM-1 antibody resulted in a complete loss of PC-induced survival advantage.^11^ To strengthen these findings regarding the requirement of NK cells for the intracranial efficacy of PC blockade, we here used a different NK cell depletion strategy and depleted NK cells using anti-NK1.1 antibody (figure 1A). Immune cells were analyzed by flow cytometry (online supplemental figure 1B) to confirm successful NK cell depletion within tumors (figure 1B), which resulted in a significantly increased intracranial tumor burden following PC blockade in comparison to mice treated in the absence of NK cell depletion (figure 1C). This involved depletion of NK cells prior to tumor initiation and throughout treatment. In contrast, we next sought to determine whether NK cells are required for ICB efficacy specifically during treatment by depleting NK cells following administration of the first dose of PC blockade (figure 1D and E). To enable a longer experimental timeline that can accommodate tumor growth in the brain and PC blockade prior to NK cell depletion, we implanted a lower number of cancer cells (figure 1D). We observed that a significant reduction in tumor burden, detected following PC blockade in comparison to IgG control group, was lost when NK cells were depleted in treated mice (figure 1F and G), demonstrating that NK cells are required for ICB-dependent control of intracranial tumor growth.

### Mechanisms of cancer cell killing in BrM following PC blockade

**Figure 2:**
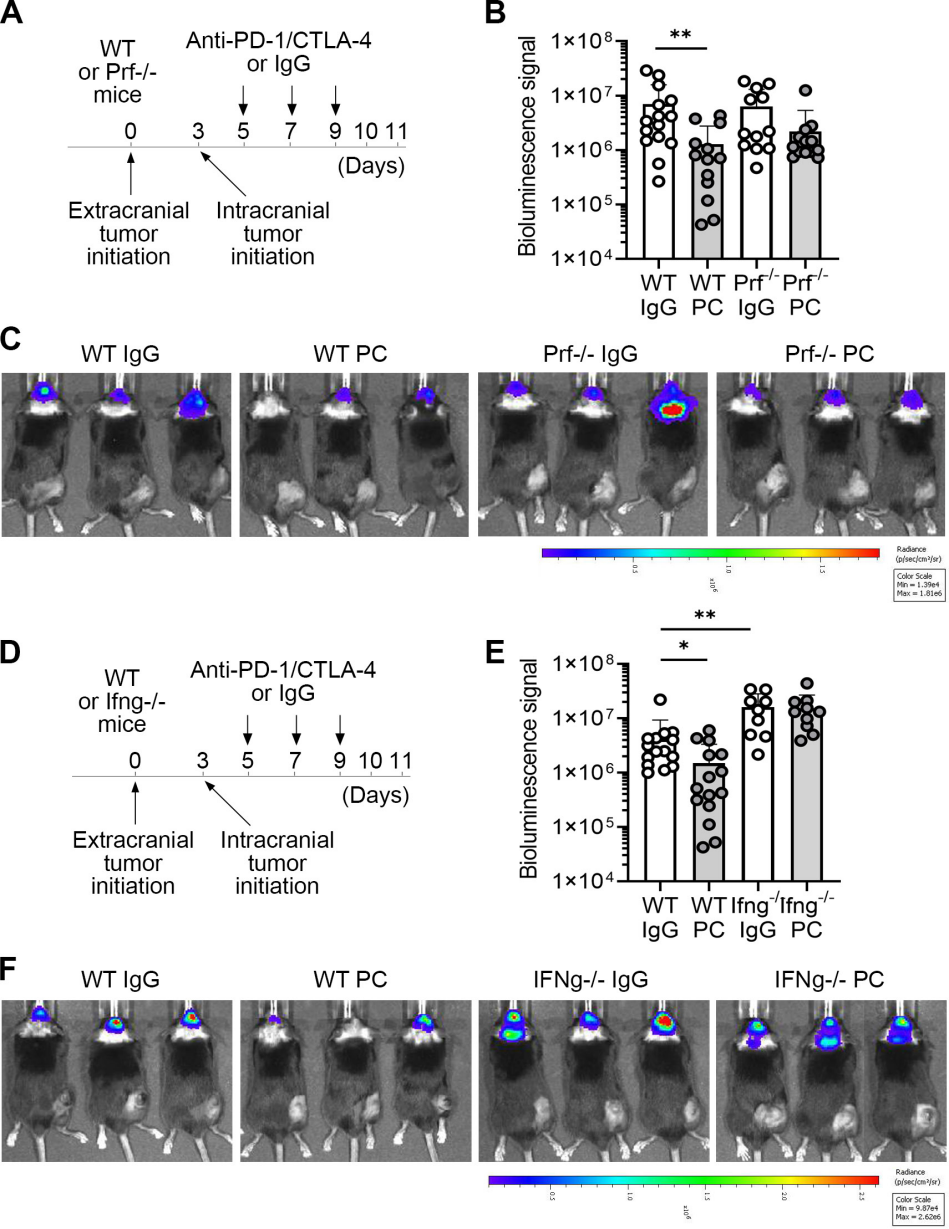
Intracranial ICB efficacy requires perforin and IFNγ. (**A**) Experimental timeline for the experiments shown in **B–C**, using wt and Prf1−/− mice. (**B**) Quantification of intracranial B16-OVA/Fluc tumor burden via bioluminescence imaging on day 10 (n=15/13/12/13 for wt IgG, wt PC, Prf1−/− IgG and Prf1−/− PC, respectively). Combined data from two independent experiments are shown. (**C**) Representative bioluminescence images for experiments shown in **B**. (**D**) Experimental timeline for the experiments shown in **E–F**, using wt and Ifng−/− mice. (**E**) Quantification of intracranial B16-OVA/Fluc tumor burden via bioluminescence imaging on day 10 (n=15/15/9/10 for wt IgG, wt PC, Ifng−/− IgG and Ifng−/− PC, respectively). Combined data from two independent experiments are shown. (**F**) Representative bioluminescence images for experiments shown in **E**. Significant differences between groups of interest in **B** and **E** were determined by unpaired Mann-Whitney test; *p≤0.05, **p≤0.01. CTLA-4, cytotoxic T-lymphocyte-associated protein 4; Fluc, Firefly luciferase; ICB, immune checkpoint blockade; IFNγ, interferon gamma; IgG, immunoglobulin G; PC, PD-1/CTLA-4; PD-1, programmed death-1; Prf, perforin; wt, wild type.

To determine the mechanisms used by lymphocytes to kill cancer cells in BrM following ICB, we investigated whether perforin (Prf) and interferon gamma (IFNγ) are required for the intracranial efficacy of PC blockade. Tumor burden in the IgG-treated control groups were comparable between Prf knock-out (ko) and wild type (wt) mice (figure 2A–C), suggesting that Prf is dispensable for the baseline control of intracranial tumor growth. However, while we observed a significant reduction in intracranial tumor burden in PC treated as compared with IgG control-treated wt mice, this effect was lost in Prf ko mice (figure 2A–C), demonstrating that Prf is required for the intracranial PC efficacy. In contrast to Prf ko, a significantly increased tumor burden was observed in the IgG control-treated IFNγ ko mice as compared with the IgG-treated wt mice (figure 2D–F). Furthermore, in IFNγ ko mice, PC therapy failed to reduce intracranial tumor burden (figure 2D–F). This indicated that IFNγ is required for the control of tumor growth in the context of ICB, as well as under baseline conditions in the absence of therapy. These data demonstrated that lymphocytes employ the perforin/granzyme system, and possibly IFNγ, to kill B16 melanoma tumors in the brain following PC blockade, with IFNγ potentially exerting other additional effects that contribute to the ICB-dependent tumor control.

**Figure 3:**
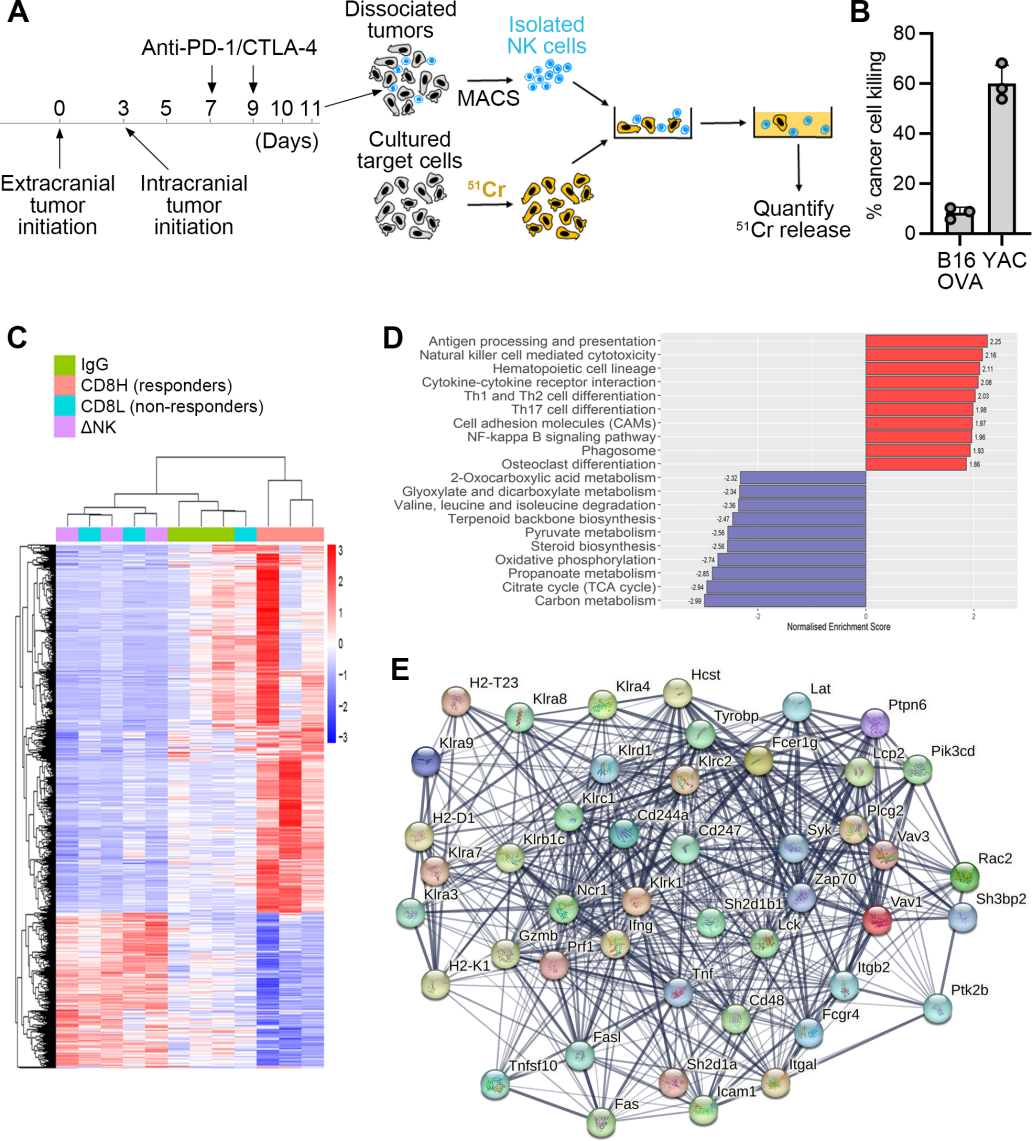
NK cell cytotoxicity and NK cell-dependent changes in tumor molecular profiles. (**A**) Experimental scheme for the isolation of NK cells from intracranial B16-OVA tumors and chromium release assay to measure NK cell-mediated killing. (**B**) Quantification of target cell killing by NK cells isolated from intracranial tumors of PC blockade treated mice, as depicted in **A**. (**C**) Unsupervised hierarchical clustering and heatmap of differentially expressed genes in bulk intracranial tumors isolated from mice on day 11 of the experimental timeline shown in **A**. NK cells were depleted using anti-Asialo-GM-1 antibody throughout the experiment. Heatmap shows the union of differentially expressed genes between all pairwise comparisons. (**D**) Top enriched KEGG terms (gene set enrichment analysis; GSEA) found to be up (red) or downregulated (blue) between CD8H intracranial tumors (responders) and the other three experimental groups, including non-responding tumors (CD8L), NK cell depleted tumors (ΔNK) and IgG control (n=3). (**E**) STRING network analysis of genes differentially expressed in KEGG term “Natural killer cell mediated cytotoxicity” from GSEA shown in **E**. Each edge in the network represents a shared association to a biological pathway between two nodes, with larger weights (increasing thickness) representing higher confidence in interaction score. ^51^Cr, ^51^Chromium; CTLA-4, cytotoxic T-lymphocyte-associated protein 4; IgG, immunoglobulin G; KEGG, Kyoto Encyclopedia of Genes and Genomes; MACS, Magnetic Activated Cell Sorting; NK, natural killer; PC, PD-1/CTLA-4; PD-1, programmed death-1.

To determine whether NK cells are directly involved in cancer cell killing in BrM, we performed an ex vivo killing assay. To this end, NK cells were isolated from intracranial B16 melanoma tumors after only two doses of PC blockade (figure 3A), to avoid therapy-induced tumor shrinkage and insufficient tumor material for downstream processing, as well as to avoid potential NK cell exhaustion. Isolated NK cells were mixed with ^51^Cr-labeled cancer cells, followed by the quantification of chromium release 4 hours later (figure 3A; online supplemental figure 2A). While brain tumor-derived NK cells efficiently killed a known NK target cell line YAC-1 (58.6±6.7%), only a low percentage of B16 cancer cell killing was observed (9.5±2.4%) (figure 3B). Because an increase in NK cell numbers following therapy could contribute to enhanced cancer cell killing despite low cytotoxicity, we next quantified NK cells in tumors by flow cytometry. In line with our previous findings,^11^ PC blockade significantly increased the abundance of intratumoral CD8+ T cells (online supplemental figure 2B). In contrast, no increase in the abundance of intratumoral NK cells was observed in PC treated versus IgG-treated control mice (online supplemental figure 2C). These findings, together with the low direct cytotoxicity of NK cells towards B16 melanoma cells pointed to CD8+ T cells as the main cytotoxic effector lymphocytes in our model and suggested that direct cancer cell killing is unlikely to be the only mechanism via which NK cells contribute to the intracranial ICB efficacy.

### Depletion of NK cells results in tumor molecular profiles reminiscent of a failed response to PC blockade

As our data suggested that direct cancer cell killing is unlikely to be the only mechanism through which NK cells mediate the intracranial efficacy of ICB, we next investigated how NK cell depletion affects the overall tumor molecular profiles. To this end, tumors were isolated from control IgG-treated mice, PC-treated mice, and mice treated with PC blockade following NK cell depletion. Notably, we observed a significant correlation between the percentage of CD8+T cells within tumors and the therapeutic efficacy, as measured by the reduction in intracranial tumor burden post-therapy (online supplemental figure 2D). This enabled us to separate mice in the PC group further into responders (characterized by high CD8+ T cell infiltration; CD8H) and non-responders (characterized by low CD8+ T cell infiltration; CD8L) (online supplemental figure 3A).

Analysis of tumors by mRNA-seq, followed by differential gene expression analysis and unsupervised hierarchical clustering, revealed significantly distinct molecular profiles of CD8H tumors responding to ICB as compared with the other three experimental groups (figure 3C). CD8H tumors were characterized by enrichment of pathways related to antigen processing and presentation, NK cell-mediated cytotoxicity, cytokine–cytokine receptor interaction, and NF-kappa B signaling pathway, as demonstrated by GSEA of KEGG pathways on differential expression between CD8H tumors and tumors from the other three groups (figure 3D**;** online supplemental figure 3D,E). Genes associated with NK cell-mediated cytotoxicity were analyzed by STRING analysis,^29^ which implements text-mining methods and hierarchical clustering of the gene network to predict protein–protein associations. These genes involved activating and inhibitory NK cell receptors, as well as the main lymphocyte effector genes, Prf1, Gzmb and IFNγ (figure 3E), which were upregulated in CD8H tumors as compared with the other 3 groups (online supplemental figure 3E). Interestingly, NK cell-depleted tumors clustered together with the CD8L tumors that failed to respond to PC, with only 40 genes being significantly differentially expressed between these two groups (online supplemental figure 3B). These genes were linked to further downregulation of pathways related to NK cell-mediated cytotoxicity and antigen processing and presentation in NK cell-depleted tumors as compared with CD8L tumors (online supplemental figure 3C). Thus, tumor molecular profiles in the absence of NK cells were very similar to tumors that failed to respond to ICB. This suggested that NK cells may be required to reshape the intracranial tumor microenvironment in a manner that supports the therapeutic response to PC blockade.

### NK cells are required for the PC blockade-induced infiltration of CD8+ T cells into BrM

**Figure 4:**
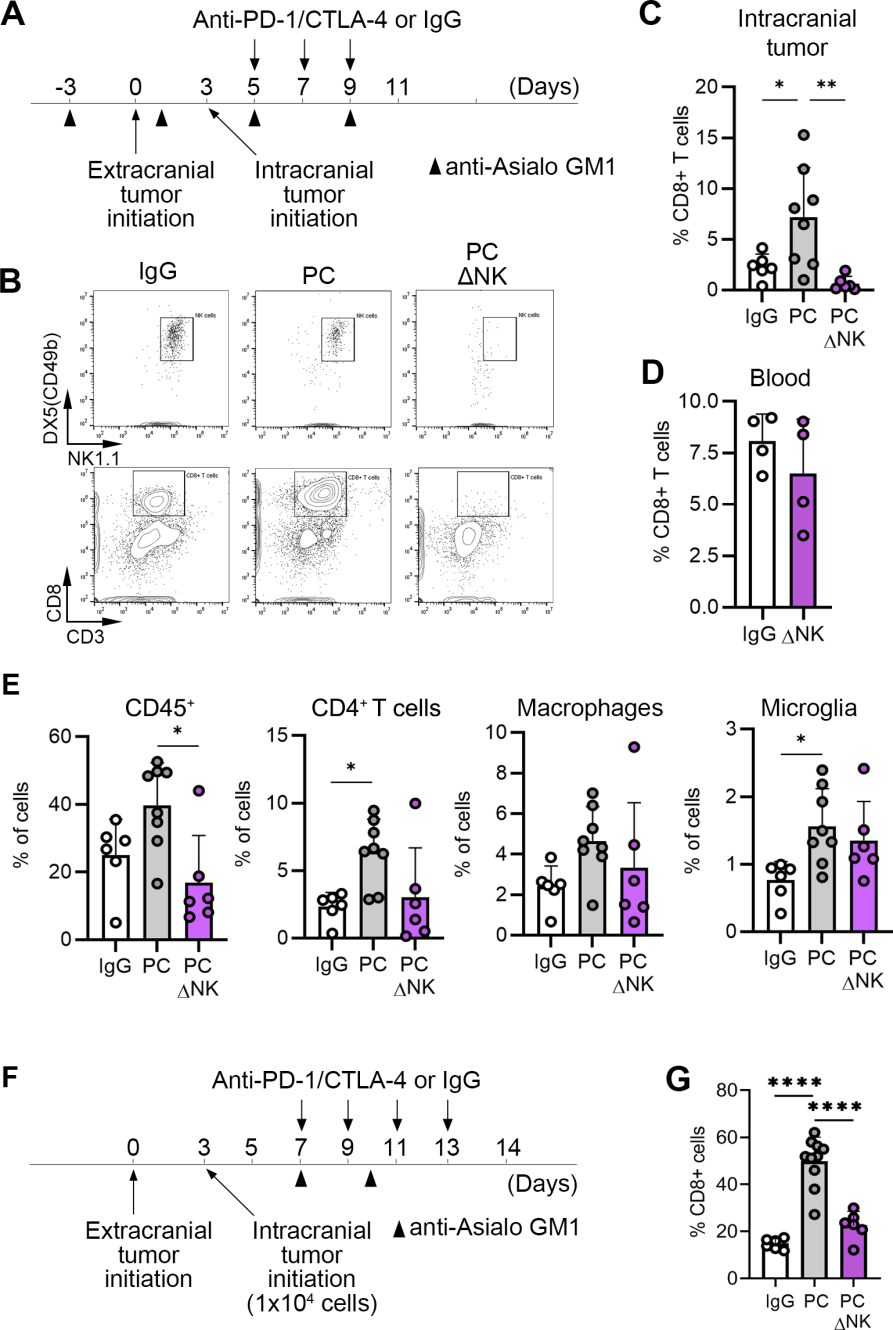
NK cells regulate the abundance of CD8+T cells in intracranial tumors. (**A**) Experimental timeline and schedule for administration of anti-Asialo-GM-1 antibodies for experiments shown in **B–E**. (**B**) Representative flow cytometry plots of NK cells and CD8+T cells in intracranial tumors at the endpoint. (**C**) Quantification of CD8+T cells in intracranial tumors (n=6/8/6 for IgG, PC and PCΔNK, respectively). One out of two representative experiments is shown. (**D**) Quantification of CD8+T cells in peripheral blood (n=4). (**E**) Quantification of CD45+cells, CD4+T cells, macrophages (CD11b+F4/80+CD45^high^) and microglia (CD11b+F4/80+CD45^low^) in intracranial tumors (n=6/8/6 for IgG, PC and PCΔNK, respectively). One out of two representative experiments is shown. (**F**) Experimental timeline and schedule for administration of anti-Asialo-GM-1 antibodies for experiment shown in **G**. (**G**) Quantification of CD8+T cells in intracranial tumors (n=6/10/6). Statistical significance in **D** was determined by unpaired two-tailed t-test. Significant differences in **C**, **E** and **G** were determined by one-way ANOVA, with exception of percentages of CD45+cells in **E**, where unpaired Kruskal-Wallis test with pairwise comparison of mean values was used; *p≤0.05, **p≤0.01, ***p≤0.001, ****p≤0.0001. ANOVA, analysis of variance; CTLA-4, cytotoxic T-lymphocyte-associated protein 4; IgG, immunoglobulin G; NK, natural killer; PC, PD-1/CTLA-4; PD-1, programmed death-1.

We next investigated how the loss of NK cells affects the composition of immune cells in the intracranial tumor microenvironment. To this end mice were treated with IgG control antibodies or PC blockade, either alone or in combination with NK cell depletion using anti-Asialo-GM-1 antibody (figure 4A). Different immune cells in tumors were quantified by flow cytometry at the endpoint (online supplemental figure 1B). While PC significantly increased the percentage of CD8+ T cells within intracranial tumors as compared with the IgG control mice, depletion of NK cells in PC treated mice reverted this phenotype and resulted in a significant reduction in intratumoral CD8+ T cells (figure 4B,C). This reduction in CD8+ T cells was local (intratumoral) rather than systemic, as no significant reduction in CD8+ T cells following NK cell depletion could be detected in the blood (figure 4D). Notably, there was no correlation between the intratumoral CD8+ T cell abundance and expression of activation markers NKp46 and CD69 on NK cells (online supplemental figure 4A,B).

Depletion of NK cells also significantly reduced the overall abundance of intratumoral immune cells (CD45+) without significantly affecting the percentages of CD4+ T cells, macrophages, and microglia (figure 4E), suggesting that CD8+ T cells are the immune cell population that is most prominently regulated by NK cells.

To confirm that the observed reduction in intratumoral CD8+ T cells is NK cell-specific, we next depleted NK cells in PC-treated mice using anti-NK1.1 instead of anti-Asialo-GM-1 antibody. In line with our previous observations, this resulted in a significant reduction in intratumoral CD8+ T cells (online supplemental figure 4C and D). Lastly, a significant reduction in the percentage of CD8+ T cells within intracranial tumors was also detected when NK cells were depleted following the first dose of PC blockade rather than throughout the experiment (figure 4F and G), demonstrating that NK cells are required for the ICB-induced increase in CD8+ T cells within intracranial tumors.

### Infiltration of CD8+ T cells into BrM is driven by chemokines

**Figure 5:**
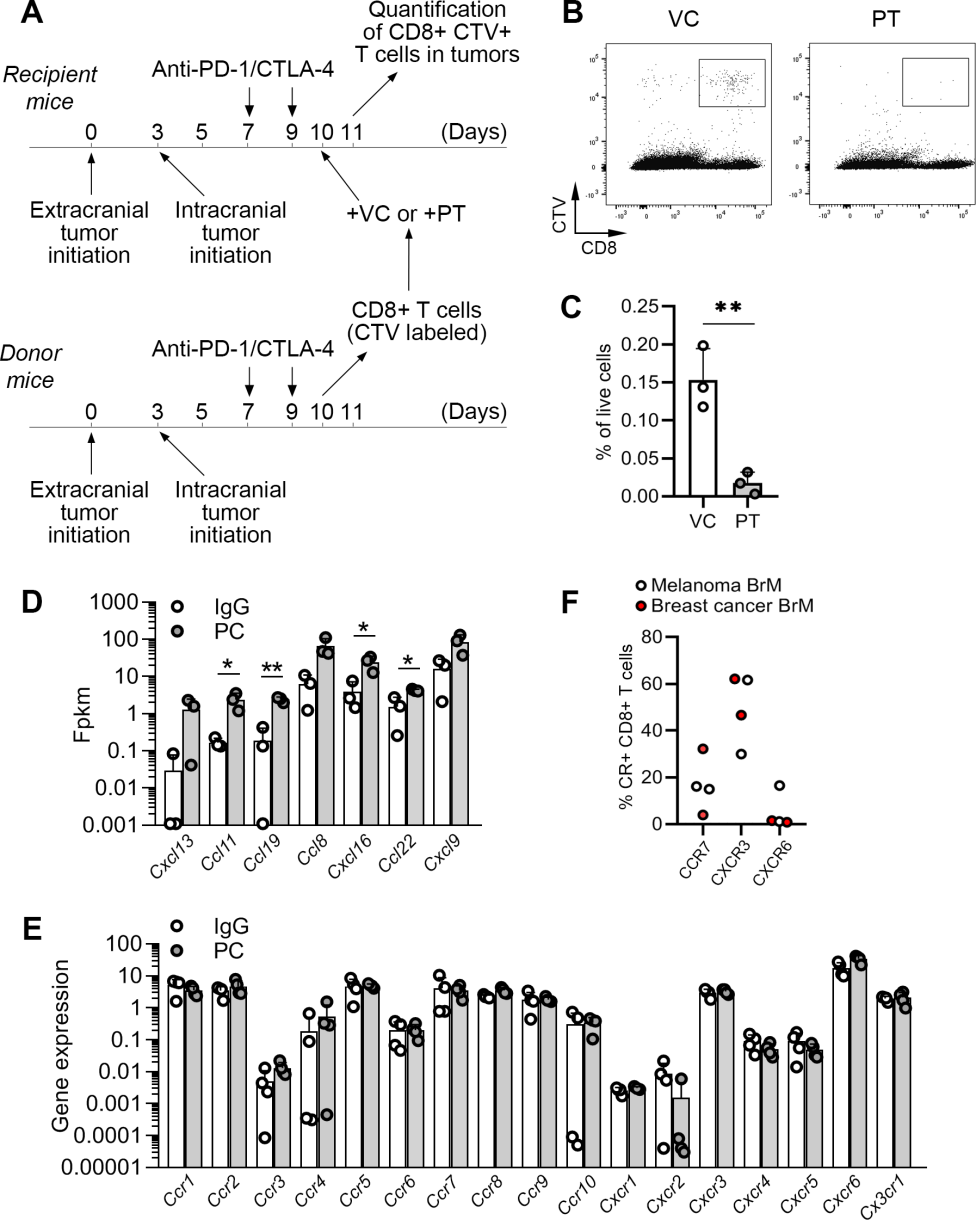
Trafficking of CD8+T cells into intracranial tumors is chemokine-dependent. (**A**) Experimental timeline for adoptive transfer of CD8+T cells. T cells isolated from donor mice were ex vivo labeled with CellTrace Violet (CTV) and treated either with pertussis toxin (PT) or vehicle control (VC) prior to their transfer into recipient mice. (**B**) Representative dot plots showing detection of adoptively transferred CTV+CD8+ T cells (following their ex vivo treatment with PT or VC) in intracranial tumors 18 hours post-transfer. (**C**) Quantification of data shown in **B**. (**D**) Analysis of chemokine gene expression in mRNA-seq data shown in figure 3. (**E**) Quantification (PCR) of chemokine receptor expression in CD8+T cells isolated from murine intracranial tumors by FACS. (**F**) Quantification of chemokine receptor expression on CD8+T cells within human brain metastases (n=2 for melanoma and n=2 for breast cancer) by flow cytometry. Statistical significances in **C** and **D** were determined by unpaired two-tailed t-test; *p≤0.05, **p≤0.01. BrM, brain metastases; CTLA-4, cytotoxic T-lymphocyte-associated protein 4; IgG, immunoglobulin G; mRNAseq, messenger RNA sequencing; PC, PD-1/CTLA-4; PD-1, programmed death-1; FACS, Fluorescence-activated cell sorting; Fpkm, Fragments Per Kilobase of transcript per Million mapped reads.

We have previously demonstrated that PC blockade in preclinical melanoma BrM models significantly increases the abundance of CD8+ T cells in intracranial tumors by enhancing their trafficking to tumors, rather than altering their proliferation and intratumoral expansion.^11^ Homing of CD8+ T cells to extracranial tumors depends on various T cell-attracting chemokines.^30^,^34^ To determine whether this is also the case in the homing of CD8+ T cells to BrM, we performed an adoptive T cell transfer experiment. CD8+ T cells isolated from tumor-bearing, PC blockade-treated mice were ex vivo labeled with CellTrace Violet (CTV), treated with vehicle (control) or Pertussis toxin to inhibit chemokine receptor-dependent signaling, and injected into tumor-bearing, PC-treated recipient mice (figure 5A). The percentage of CTV+CD8+ T cells within tumors was quantified by flow cytometry 18 hours later (figure 5B). Pertussis toxin treatment resulted in a strong and statistically significant reduction in the percentage of adoptively transferred CD8+ T cells within intracranial tumors (figure 5C), confirming that CD8+ T cell homing to BrM is chemokine dependent.

In line with enhanced infiltration of CD8+T cells into intracranial tumors following PC blockade, we observed an increase in gene expression for several T cell attracting chemokines in intracranial tumors of PC treated as compared with the control mice, including *Ccl22*, *Ccl19* and *Cxcl16,* and a tendency for *Cxcl9* (figure 5D).

*Ccl22* is known to attract regulatory T cells (Tregs) and T helper 2 cells,^35 36^ and its upregulation was in line with our previous observations of PC-induced increase in Tregs in intracranial tumors in the two-site BrM model.^11^ However, CD4+ T cells were not functionally involved in the intracranial efficacy of PC blockade,^11^ and therefore we here focused on the other three chemokines. To determine whether cognate chemokine receptors are expressed on CD8+ T cells infiltrating intracranial tumors, the latter were isolated from PC-treated and control mice by fluorescence-activated cell sorting (FACS), followed by quantification of chemokine receptor gene expression by quantitative PCR (qPCR). Genes for chemokine receptors *Ccr7*, *Cxcr3* and *Cxcr6*, which interact with the respective T cell-attracting chemokines CCL19, CXCL9 and CXCL16, were highly expressed in CD8+T cells within intracranial tumors, with comparable expression levels in PC treated and control mice (figure 5E). Moreover, these chemokine receptors were also expressed on CD8+T cells infiltrating human BrM, as quantified by flow cytometry, with the highest expression being detected for CXCR3 (figure 5F; online supplemental figure 5A).

To assess the functional involvement of CXCR3 in CD8+T cell homing to BrM, we performed an adoptive T cell transfer experiment in which CTV-labeled CD8+ T cells were ex vivo pretreated with CXCR3 blocking antibody or IgG control prior to their transfer into recipient mice. We observed a tendency towards a reduced homing of CD8+ T cells following CXCR3 blockade, but this did not reach statistical significance in comparison to the control group (online supplemental figure 5B). Together with the observed significant reduction in CD8+ T cell homing to BrM following pertussis toxin treatment, this suggested that multiple chemokines, in addition to CXCL9/10, may be involved.

### NK cells are required for the upregulation of CD8+ T cell attracting chemokines and vascular T cell entry receptors in BrM following ICB

**Figure 6:**
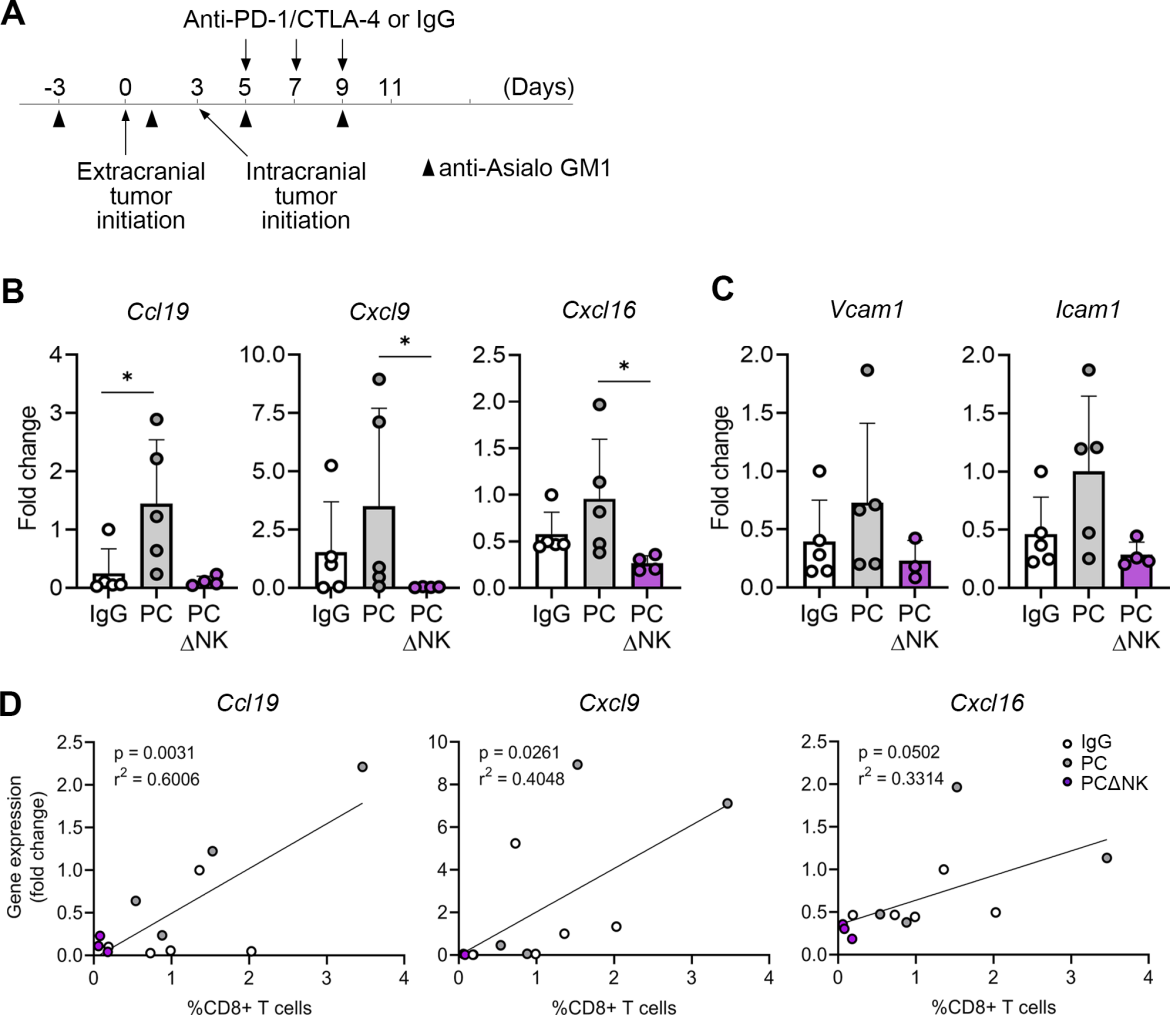
NK cells are required for the upregulation of T cell-attracting chemokines and vascular T cell entry receptors in intracranial tumors following PC blockade. (**A**) Experimental timeline and schedule for administration of anti-Asialo-GM-1 antibodies for experiments shown in **B–D**. Quantification of gene expression for T cell-attracting chemokines (**B**) and vascular T cell entry receptors (**C**) in intracranial tumors by qPCR (n=5/5/4 for IgG, PC and PCΔNK, respectively). (**D**) Correlation between the expression of chemokine genes and percentage of CD8+T cells in intracranial tumors. The fold change in **B–C** was calculated according to the ΔΔCt method (fold change=2-(ΔΔCt)) to provide relative comparison in gene expression between samples. Normalization to the expression levels of the housekeeping gene GAPDH was performed during the calculation of ΔΔCt values for each gene in each sample by subtracting the Ct value of GAPDH from the Ct value of the chemokine genes of interest. Statistical significances were determined by unpaired Kruskal-Wallis test with pairwise comparison of mean values (**B**) and one-way ANOVA (**C**); *p≤0.05. ANOVA, analysis of variance; Ct, cycle threshold; CTLA-4, cytotoxic T-lymphocyte-associated protein 4; IgG, immunoglobulin G; NK, natural killer; PC, PD-1/CTLA-4; PD-1, programmed death-1; qPCR, quantitative PCR.

Because our data demonstrated that NK cells are important for the infiltration of CD8+ T cells in BrM, and this was chemokine dependent, we asked the question of whether NK cells are required for the intratumoral expression of T cell-attracting chemokines. Tumors were isolated from control mice, PC-treated mice, and PC-treated mice in which NK cells were depleted using anti-Asialo-GM1 antibody (figure 6A). Gene expression was quantified by qPCR. We observed a significant downregulation in *Cxcl9* and *Cxcl16* gene expression, and a tendency towards the reduced expression of *Ccl19*, in NK cell-depleted PC treated mice as compared with the mice treated with PC in the presence of NK cells (figure 6B). Moreover, there was a tendency towards reduced gene expression of vascular T cell entry receptors *Icam1* and *Vcam1* in NK cell-depleted mice (figure 6C). The proportion of CD8+ T cells within intracranial tumors correlated significantly with the gene expression levels of *Ccl19* and *Cxcl9*, and there was a tendency towards positive correlation with *Cxcl16* expression levels (figure 6D). In summary, these data provide evidence that NK cells are likely regulating homing of CD8+ T cells to BrM by promoting intratumoral expression of T cell-attracting chemokines and vascular T cell entry receptors.

In summary, our data demonstrate that NK cells play an important role in the efficacy of combined PC blockade that goes beyond direct cancer cell killing and involves reshaping of the tumor microenvironment at the cellular and molecular level as well as enhanced recruitment of CD8+ T cells.

## Discussion

While CD8+ T cells have been widely studied in the context of ICB, our understanding of how NK cells contribute to ICB efficacy is overall still limited, and the role of NK cells in ICB in BrM remains unexplored. Our study addresses this knowledge gap and provides evidence for a major role of NK cells in shaping molecular and cellular profiles of BrM in a manner that supports the activity of immune checkpoint inhibitors, including by supporting enhanced infiltration of effector CD8+ T cells.

Even though perforin was required for intracranial ICB efficacy in our BrM model, we observed only low cytotoxic activity of intracranial tumor-derived NK cells towards B16 melanoma cells. The latter has been previously shown to express MHC-I and to display a low susceptibility to direct NK cell-mediated killing.^37^ In line with this, we observed MHC-I expression by the majority of CD45- cells within intracranial B16 tumors (data not shown). The use of an MHC-I positive model with low susceptibility for direct killing by NK cells in our study allowed for exposure of cytotoxicity-unrelated roles of NK cells in intracranial ICB activity. While a tendency towards reduced abundance was observed for several intratumoral immune cell populations following NK cell depletion, CD8+ T cell abundance was affected most severely, and this was the only immune cell population that was statistically significantly reduced. Because ICB-induced increase in intratumoral CD8+ T cells was chemokine-dependent and the expression of T cell attracting chemokines in tumors was impaired following NK cell depletion, our data strongly suggest that NK cells are responsible for the recruitment of CD8+ T cells by upregulating intratumoral chemokine expression. In our analysis we focused on *Ccl19*, *Cxcl9* and *Cxcl16*, because these chemokines were upregulated in BrM following PC blockade and have been previously implicated in the attraction of T cells to tumors.^30^,^34^ The three cognate chemokine receptors CXCR3, CCR7 and CXCR6 were highly expressed on tumor-infiltrating CD8+ T cells in our preclinical model. We also observed a variable expression of these chemokine receptors on CD8+ T cells within human BrM samples, suggesting a potential interpatient heterogeneity and potential variability in chemokines and/or chemokine combinations driving CD8+ T cell infiltration into melanoma BrM. While we also observed upregulation of the Treg-attracting chemokine *Ccl22* following PC blockade, depletion of CD4+ T cells in this model in our previous study had no effect on the intracranial efficacy of PC blockade,^11^ suggesting that Tregs do not play a major role in this context.

Notably, NK cells could be recruiting CD8+ T cells into tumors directly or indirectly. NK cells have been previously shown to recruit dendritic cells via Ccl5, Xcl1 and Flt3l,^38 39^ and dendritic cells, as well as macrophages, are known to express T cell-attracting chemokines Cxcl9 and 10.^31 40 41^ Thus, the recruitment of CD8+ T cells to tumors by NK cells could be indirect.

Expression of PD-1 on tumor-associated murine and human NK cells is well established,^14^,^17^ and there is also some evidence for CTLA-4 expression on NK cells.^18^,^20^ Both PD-1 and CTLA-4 on NK cells have been shown to inhibit their cytotoxic activity towards cancer cells and their cytokine production.^14^,^20^ Thus, anti-PD-1 and anti-CTLA-4 in our model are likely acting on NK cells directly. Alternatively, anti-CTLA-4 could also activate NK cells indirectly via the depletion of Tregs.^42^ One thing that remains unclear is where the interaction between therapeutic antibodies and NK cells takes place, especially considering potentially limited access of therapeutic antibodies to the brain. We have previously shown that in a two-site BrM model, PC blockade activates and expands CD8+ T cells extracranially, suggesting that therapeutic antibodies don’t necessarily need to access the brain for their efficacy. Whether this is also the case for NK cells remains to be determined in future studies.

We observed that tumor molecular profiles following PC blockade were very similar between tumors that failed to respond to therapy and those that were depleted of NK cells. Pathways related to antigen processing and presentation, NK cell-mediated cytotoxicity, and cytokine–cytokine receptor interaction were downregulated in these tumors as compared with tumors that responded to PC blockade. This suggested that NK cells are required for the establishment of intracranial tumor molecular profiles that support response to ICB. Interestingly, NK cells have been previously shown to be required for early tumor polarization towards cancer inhibitory inflammation and for recruitment of CD8+ T cells during natural antitumor immunity observed specifically in cyclooxygenase-deficient tumors.^43^ Moreover, NK cells were also involved in the reshaping of the tumor microenvironment into one responsive to ICB through sensitization therapy consisting of IFNγ, anti-IL-10 and TLR3 agonist poly-IC in subcutaneous tumor models.^21^ Overall, these studies together with our data suggest that NK cells promote tumor molecular profiles supportive of antitumoral immune responses and ICB activity in various contexts. These findings have potentially important therapeutic implications in the context of ICB, as well as in the context of adoptive T cell and NK cell therapies. For example, intraperitoneal injection of induced pluripotent stem cell (iPSC)-derived NK cells have been shown to enhance the recruitment of both CD4+ and CD8+ T cells to the peritoneum in tumor-naïve mice, and iPSC-derived NK cells also enhanced the efficacy of PD-1 blockade in tumor-bearing mice in ovarian cancer model.^44^ Thus, combining NK cell therapies with ICB in the context of BrM may be a good strategy to improve therapeutic efficacy. Further studies into molecular characteristics of NK cells and how these could be manipulated to achieve the best therapeutic vehicles will be required to inform such strategies further.

### Declarations

#### Ethics approval and consent to participate

Consent from all patients donating BrM tissue was obtained. All animal procedures were approved by the Animal Welfare and Ethical Review Committee (University of Leeds) and performed under the approved UK Home Office project license in accordance with the Animal (Scientific Procedures) Act 1986 and the UK National Cancer Research Institute Guidelines for the welfare of animals.^45^

## supplementary material

10.1136/jitc-2024-009522 online supplemental file 1

## Data Availability

Data are available in a public, open access repository.
