# Supplementary material for: Natural killer cells are required for the recruitment of CD8+ T cells and the efficacy of immune checkpoint blockade in melanoma brain metastases
Source: J Immunother Cancer. 2024 Nov 17;12(11):e009522. doi: 10.1136/jitc-2024-009522 (PMC11574513; doi:10.1136/jitc-2024-009522)
Supplement: online supplemental file 1 [file jitc-12-11-s001.pdf]

Supplemental methods

Tissue dissociation and flow cytometry

Mouse blood was collected from the tail vein and red blood cells were lysed prior to analysis. Mice were perfused with saline prior to tumor isolation. Both mouse and human tumor tissue was mechanically disrupted and subsequently enzymatically dissociated as previously described (23). Prior to surface antigen staining, murine samples were blocked with 10% rat serum and mouse FcRγ blocking reagent (Miltenyi), and human samples were blocked with 10% mouse serum and human FcRγ blocking reagent (Miltenyi). Samples were analyzed on Cytoflex LX (Beckman Coulter) flow cytometer. Data were quantified using FlowJo™ software (BD) or CytExpert V.2.3 software (Beckman Coulter). The gates were set based on fluorescence minus one (FMO) controls. CD8+ T cells were sorted from dissociated murine intracranial tumor tissue using BD Influx (BD Biosciences) cell sorter. Anti-mouse CD45 (30-F11), CD8b (YTS156.7.7), NK1.1 (PK136), NKp46 (29A1.4) and CD69 (H1.2F3) were from Biolegend. Anti-mouse CD3ε (145-2C11), CD4 (GK1.5) and CD49b (DX5) were from Miltenyi. Anti-mouse CD11b (M1/70) was from BD Bioscience. Anti-mouse F4/80 (Cl:A3-1) was from BioRad. Anti-human CD45 (H130), Anti-human CD8 (SK1), anti-human CCR3 (5E8), anti-human CCR7 (G043H7), anti-human CXCR3 (G025H7), anti-human CXCR5 (J252D4), and anti-human CXCR6 (K041E5) were from Biolegend. Anti-human CD3 (BW264/56) was from Miltenyi. Antibodies used to analyze human NK cells in patient BrM were anti-CD14 (M5E2), anti-CD15 (W6D3), anti-D163 (GHI/61), anti-CD3 (UCHT1), anti-CD19 (HIB19), anti-CD45 (HI30), anti-CD56 (HCD56), anti-NKp46 (9E2), and anti-CD69 (FN50), and were all obtained from Biolegend.

Adoptive T cell transfer

CD8+ T cells were isolated from the spleens and inguinal, axial, and mesenteric lymph nodes of tumor bearing, PC treated mice on day 8 post-intracranial B16/Fluc cancer cell implantation via magnetic cell sorting using CD8 T cell isolation kit (Milteny). Isolated T cells were *ex vivo* labelled with Cell Trace Violet (CTV) (Invitrogen) and subsequently treated with vehicle control or Pertussis

toxin (Sigma) at 100 ng/mL for 1 hour at 37°C, while in CXCR3 blocking experiments CTV-labelled T cells were treated with PBS or anti-CXCR3 antibody (CXCR3-173; Bio-X-cell) at 200 µg/mL for 1 hour at 37°C. The cells were subsequently washed with PBS and injected i.v. into the tail vein of tumor bearing, PC treated recipient mice ($4x10^6$ cells per mouse in 200 uL), on day 9 post-intracranial implantation of B16/Fluc cancer cells.

Quantification of gene expression

For chemokine receptor gene expression analysis, Smart-seq2 was utilized due to low levels of RNA extracted from FACS sorted CD8+ T cells. Briefly, reverse transcription, PCR pre-amplification and PCR purification were performed as per (44).

RT-PCR primers were designed using Primer3 software v0.4.0. The following primers were used: Ccr1_Fwd: 5’-gccctcatttcccctacaa-3’, Ccr1_Rev: 5’-cggctttgaccttcttctca-3’, Ccr2_Fwd: 5’-tgtgggacagaggaagtgg-3’, Ccr2_Rev: 5’-ggaggcagaaaatagcagca-3’, Ccr3_Fwd: 5’-gacatgtgctggccagttga-3’, Ccr3_Rev: 5’-ggccggtagaaaggggtttc-3’, Ccr4_Fwd: 5’-ccaaacccaaatcaaaacca-3’, Ccr4_Rev: 5’-gagaaggaaagagaaggcgtaaa-3’, Ccr5_Fwd: 5’-tttgttcctgccttcagacc-3’, Ccr5_Rev: 5’-ttggtgctctttcctcatctc-3’, Ccr6_Fwd: 5’-tcttcacccctttgctgttt-3’, Ccr6_Rev: 5’-gctctgtgcctcttggagtt-3’, Ccr7_Fwd: 5’-attgctgctgagggaagag-3’, Ccr7_Rev: 5’-acttttggctgtcgttttgg-3’, Ccr8_Fwd: 5’-tccacgctgtctatgccatc-3’, Ccr8_Rev: 5’-tgcagggacgtgaggaaaag-3’, Ccr9_Fwd: 5’-gagtcttgctcccaatccac-3’, Ccr9_Rev: 5’-taggttcccaccatccaaac-3’, Ccr10_Fwd: 5’-cctcaatccggtgctttatg-3’, Ccr10_Rev: 5’-tttctcaacgtcccccctactt-3’, CXCR1_Fwd: 5’-tgtcccttctgagcttgctg-3’, CXCR1_Rev: 5’-ccaagaagggcagggtcaat-3’, CXCR2_Fwd: 5’-tgtctgctcccttccatctt-3’, CXCR2_Rev: 5’-ccatttcctctcctccacct-3’, CXCR3_Fwd: 5’-gcctcaatccgctgctctat-3’, CXCR3_Rev: 5’-cacacagggatggctgagtt-3’, CXCR4_Fwd: 5’-ctacagcagcgttctcatcct-3’, CXCR4_Rev: 5’-cttttcagccagcagtttcct-3’, CXCR5_Fwd: 5’-acctcccatcctaaccagct-3’, CXCR5_Rev: 5’-ttctgggaggagagtctggg-3’, CXCR6_Fwd: 5’-ggcagataatgttcagggctct-3’, CXCR6_Rev: 5’-actgcttgcccactgaagaa-3’, CX3CR1_Fwd: 5’-acccctttatctacgcctttg-3’, CX3CR1_Rev: 5’-ctgtcctgcctgctcctct-3’, Tbp_Fwd:: 5’-gagttgcttgctctgtgctg-3’,

Tbp_Rev: 5’-atactgggaaggcggaatgt-3’. cDNA samples, forward and reverse primers and SYBR Green qPCR Master Mix (ThermoFisher) were loaded into 384-well plates and RT-PCR was performed using a QuantStudio™ 7 Flex Real Time PCR System (ThermoFisher). Chemokine receptor gene expression levels were normalized to TATA-binding protein (Tbp) gene expression.

For chemokine and vascular entry receptor gene expression analysis, cDNA was generated directly from isolated RNA using SuperScript™ III Reverse Transcriptase (Invitrogen) as per manufacturer’s instructions. Predesigned TaqMan™ Real-Time PCR Assays (Taqman™ probe) (ThermoFisher) were used: Ccl19: Mm00839966_g1, Cxcl9: Mm01345157_m1, Cxcl16: Mm01248660_g1, Vcam1: Mm01320973_m1, Icam1: Mm00516025_g1. cDNA samples, Taqman™ probes and Taqman™ Fast Advanced Master Mix were loaded into 384-well plates and RT-PCR performed as described above.

Supplemental Figure 1

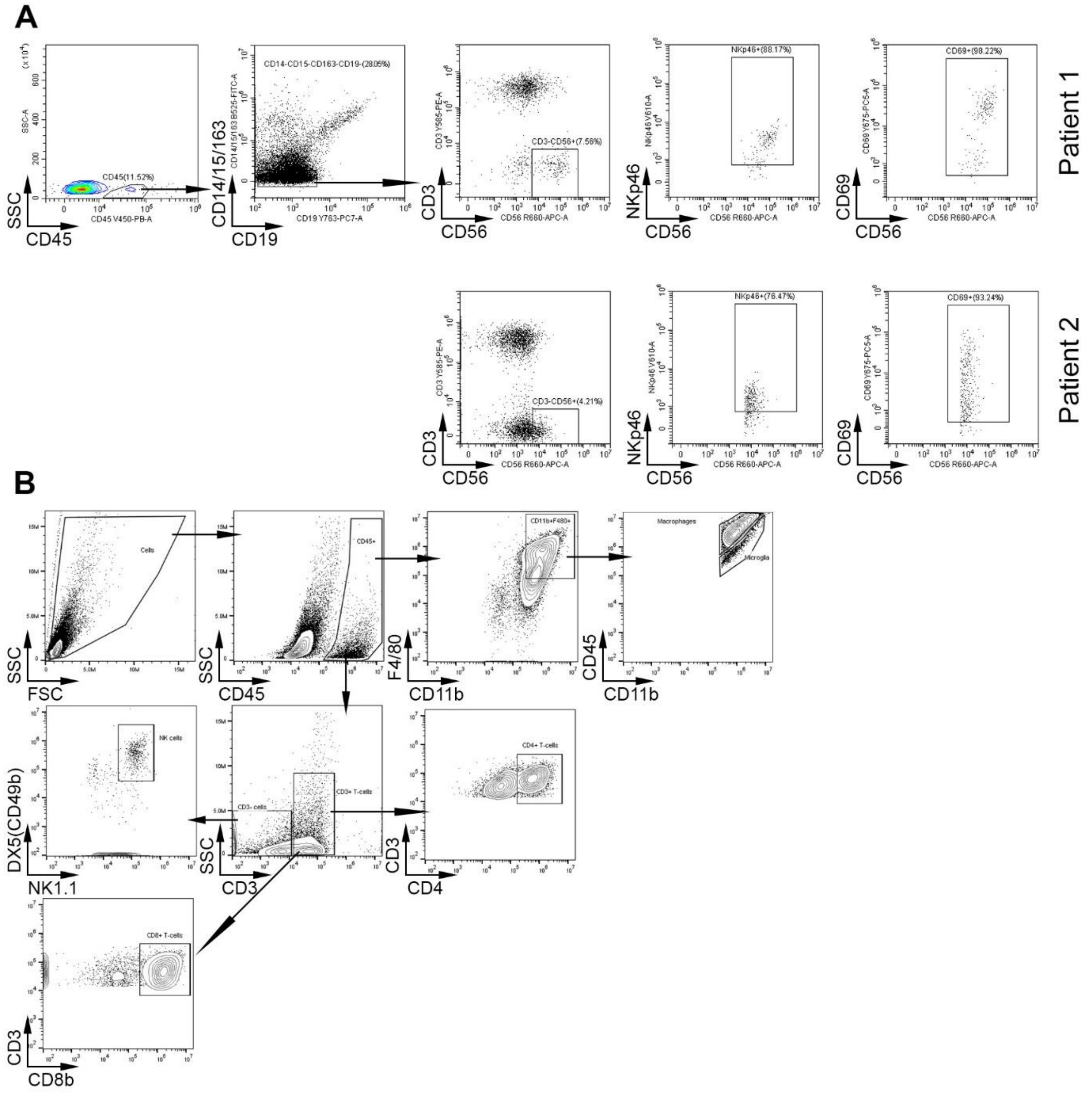

Supplemental Figure 1. Quantification of immune cell populations in human BrM and in murine intracranial tumors. (A) Gating strategy and flow cytometry plots showing the quantification of NK cells in human BrM tissue. CD45+ cells were gated on CD14-CD15-CD163- and CD19- cells to exclude myeloid cells and B cells, followed by gating on CD3-CD56+ NK cells. Quantification of NKp46+ and CD69+ NK cells was performed within the CD3-CD56+ population. **(B)** Gating strategy and representative flow cytometry plots for quantification of different immune cell populations in intracranial tumors in the in vivo B16 two-site model are shown.

Supplemental Figure 2

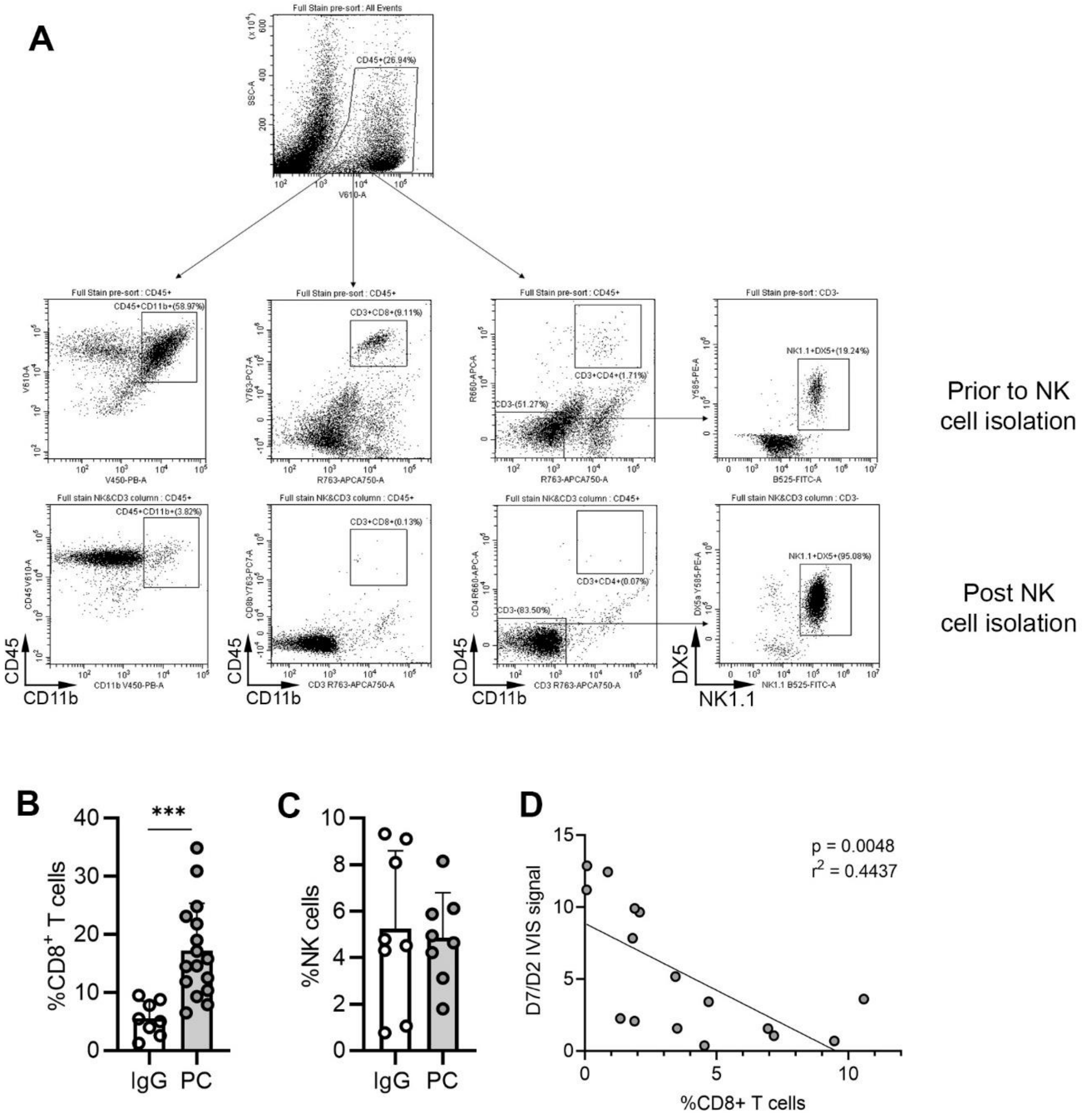

Supplemental Figure 2. Purity of isolated NK cells and quantification of CD8+ T cells and NK cells in intracranial tumors. (A) Analysis of NK cell purity following their isolation from intracranial tumors by MACS. Flow cytometry plots of samples pre/post purification are shown. **(B)** Quantification of CD8+ T cells in intracranial tumors by flow cytometry (n = 8/16). One out of three representative experiments is shown. **(C)** Quantification of NK cells in intracranial tumors by flow cytometry (n = 8/8). One out of three representative experiments is shown. **(D)** Correlation between percentage of CD8+ T cells in intracranial tumors (combined IgG and PC group) and intracranial therapeutic efficacy, expressed as a ratio in IVIS bioluminescence signal (tumor burden) between days 7 and 4

post-intracranial tumor initiation (following experimental timeline shown in Figure 1A). One out of 2 independent experiments is shown. Statistical significance in A and B was determined by unpaired two-tailed T-test; *** $p \leq 0.001$.

Supplemental Figure 3

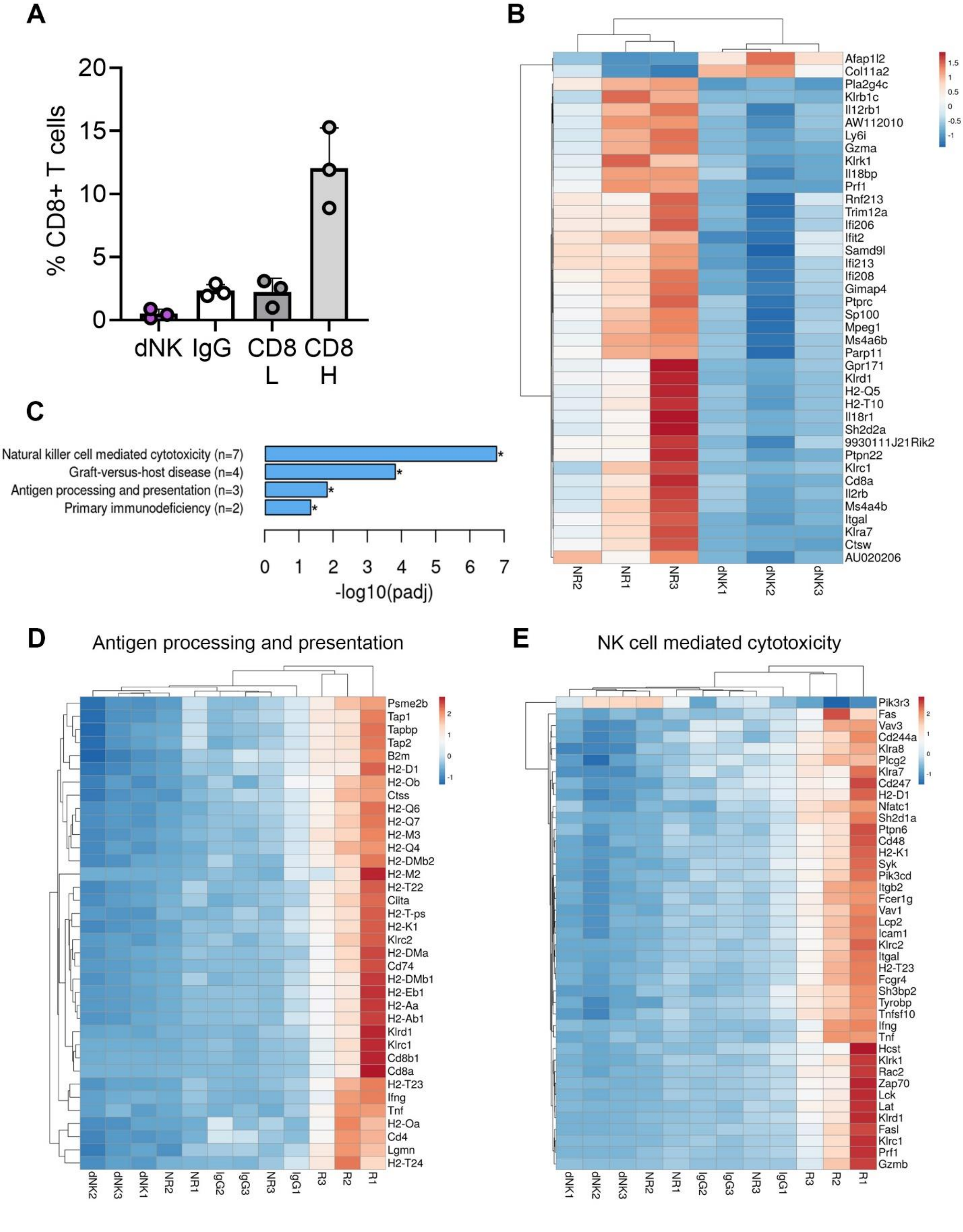

Supplemental Figure 3. Analysis of tumor molecular profiles. (A) Abundance of CD8+ T cells in intracranial tumors from individual experimental groups included in mRNAseq analysis, including IgG control and 3 groups receiving PC blockade: responders (CD8H), non-responders (CD8L) and NK cell

depleted group (dNK). **(B)** Heat map showing genes differentially expressed in intracranial tumors between CD8L (non-responder; NR) and dNK group. **(C)** KEGG terms (GSEA) that were significantly enriched between CD8L and dNK group. Heat maps of differentially expressed genes withing KEGG term “Antigen processing and presentation” **(D)** and “Natural killer cell mediated cytotoxicity” **(E)** shown in Figure 3D. Experimental groups included CD8H (responders; R), CD8L (non-responders; NR), NK cell depleted group (dNK) and IgG control group.

Supplemental Figure 4

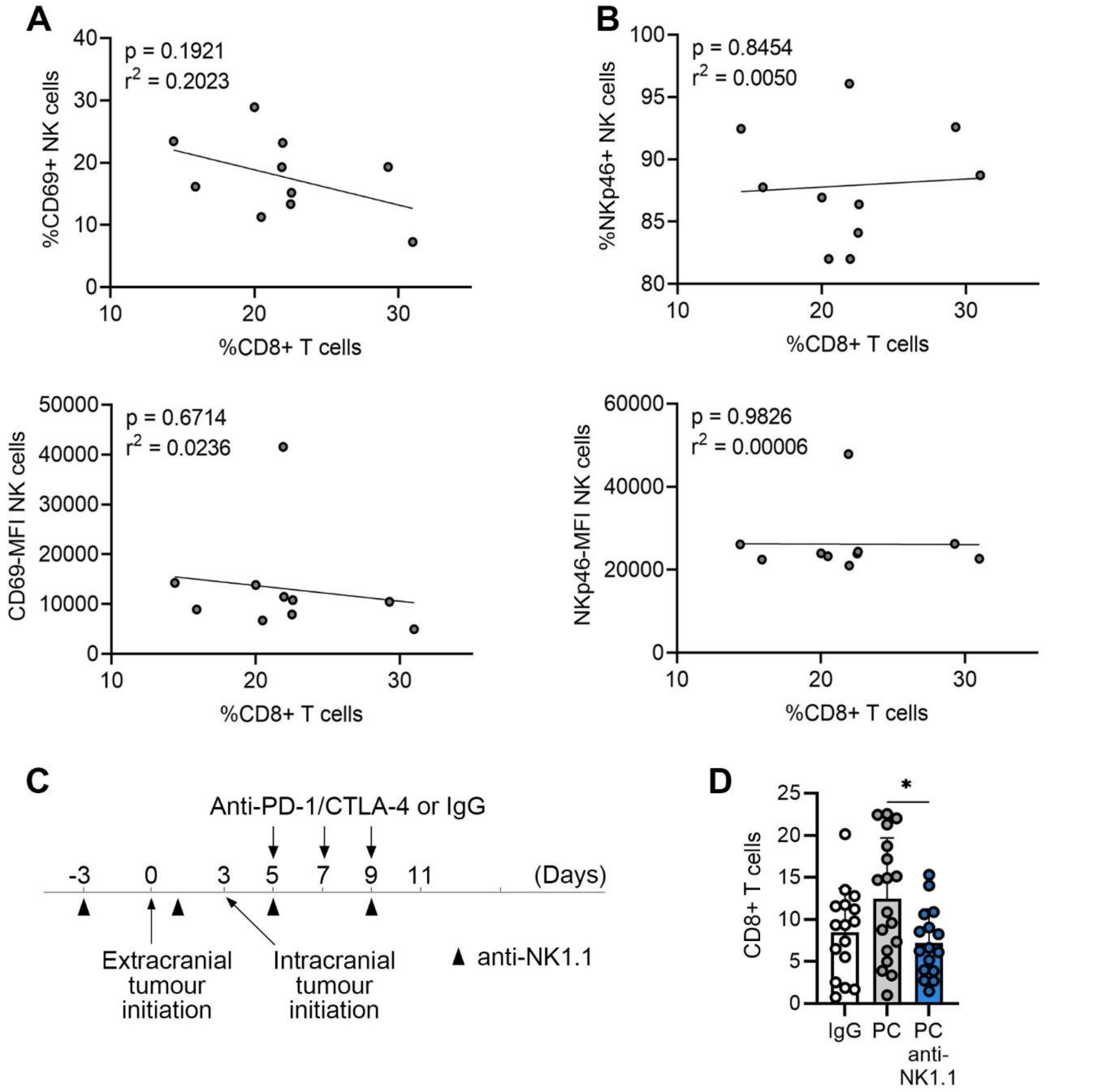

Supplemental Figure 4. Lack of correlation between CD8+ T cell abundance and expression of NK cell activation markers, and impact of NK cell depletion (anti-NK1.1 antibody) on the abundance of CD8+ T cells in intracranial tumors. (A) Correlation between the abundance of intra-tumoral CD8+ T cells and percentage CD69+ NK cells (top) or CD69 MFI in NK cells (bottom), as quantified by flow cytometry. **(B)** Correlation between the abundance of intra-tumoral CD8+ T cells and percentage NKp46+ NK cells (top) or NKp46 MFI in NK cells (bottom), as quantified by flow cytometry. **(C)** Experimental timeline and schedule for administration of anti-NK1.1 antibodies for the experiments shown in D. **(D)** Quantification of CD8+ T cells in intracranial tumors (n = 16/18/16 for IgG, PC and PC

anti-NK1.1, respectively). Pooled data from two independent experiments are shown. Statistical significance in B was determined by one-way ANOVA; * $p \leq 0.05$.

Supplemental Figure 5

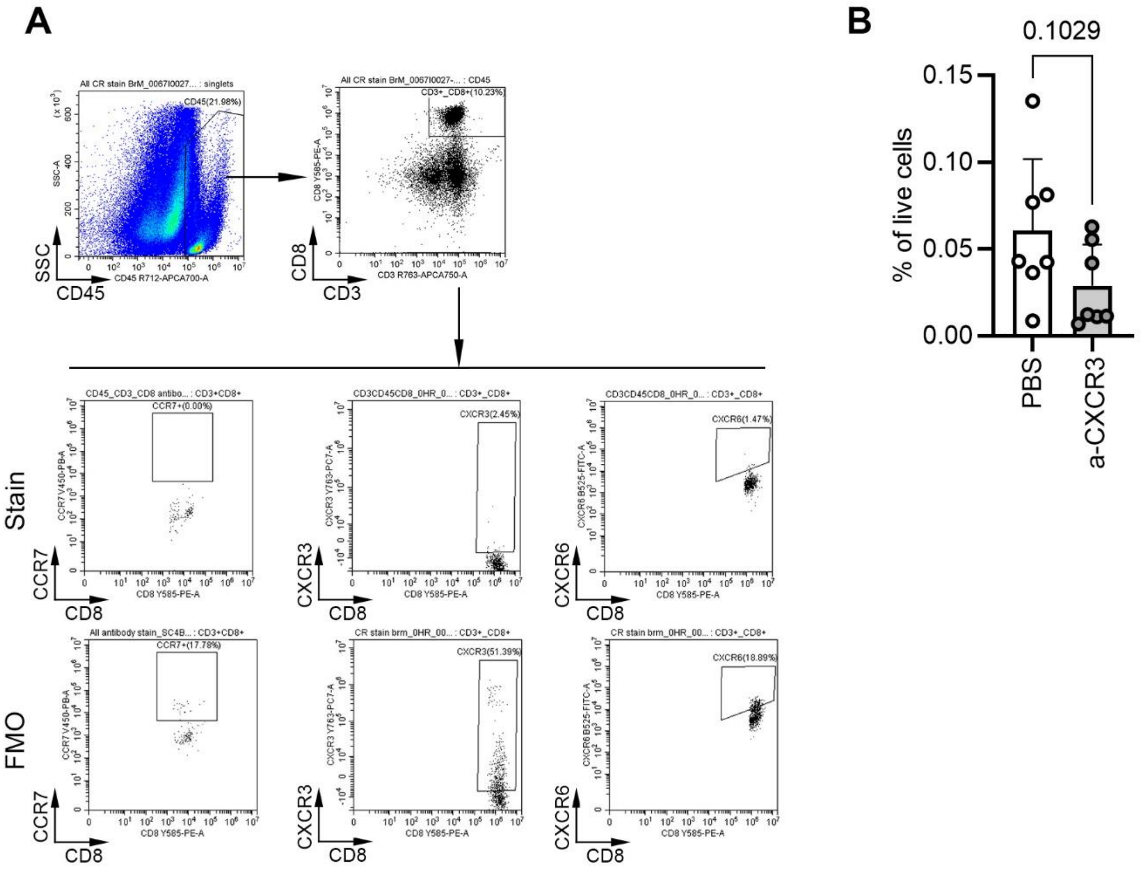

Supplemental Figure 5. Chemokine receptor expression on CD8+ T cells in human brain metastases tissue. (A) Gating strategy and representative flow cytometry plots for quantification of chemokine receptor expression on CD8+ T cells within human brain metastases tissue by flow cytometry. **(B)** Adoptive transfer was performed as illustrated in Figure 5A, with CTV+ CD8+ T cells being *ex vivo* treated with PBS or CXCR3 blocking antibody prior to their transfer into recipient mice. Quantification of CTV+ CD8+ T cells in intracranial tumors and spleen tissue was performed by flow cytometry at 18 hours post-transfer. Percentage of CTV+ CD8+ T cells within intracranial tumors was normalized to the percentage within the spleen, to account for inter-mouse variability. Data was excluded where a low frequency of CTV+ CD8+ T cells were observed in the spleen, indicative of poor i.v. injection of adoptively transferred T cells. Pooled data from two independent experiments is shown. Statistical significance was analysed by unpaired Kruskal-Wallis test with pairwise comparison of mean values.
